# Setting the lifespan clock: anti-ageing interventions during development

**DOI:** 10.1098/rstb.2025.0343

**Published:** 2026-11-12

**Authors:** Xusheng Hao, Ye Tian

**Affiliations:** ^1^ Institute of Genetics and Developmental Biology CAS, Beijing, People's Republic of China; ^2^ University of the Chinese Academy of Sciences, Beijing, People's Republic of China

**Keywords:** early-life interventions, longevity, ageing trajectories, epigenetics reprogramming, metabolic plasticity

## Abstract

Ageing trajectories may be established long before the onset of functional decline, potentially during early developmental stages. This review highlights evidence that transient developmental interventions—encompassing metabolic, pharmacological, microbial, and physiological approaches—may confer lasting benefits for longevity and healthspan across species. The long-term effects are proposed to be mediated by systemic epigenetic and metabolic reprogramming, establishing a primed state of enhanced stress resistance and homeostatic capacity that persists into adulthood. Critically, this developmental programming often operates within a narrow window of high plasticity; the same interventions are often ineffective or even harmful when administered later in life. These findings underscore the importance of timing in geroscience and suggest that understanding early-life plasticity provides a valuable conceptual framework for exploring future strategies to delay ageing.

This article is part of the theme issue ‘Developmental circuits in ageing’.

## 1. Introduction

Ageing is a complex biological process characterized by progressive functional decline and increased susceptibility to disease [1–3]. While geroscience has traditionally focused on targeting these processes in later life, emerging evidence suggests that ageing trajectories are established much earlier. Research into biological clocks spanning the full life cycle demonstrates that developmental and early-life periods critically influence the long-term pace of ageing [4]. This paradigm shift highlights the need to integrate developmental biology into ageing research to fully understand how ageing is programmed.

Human epidemiological studies provide compelling evidence that early-life conditions correlate with adult health patterns. These observations align with the Developmental Origins of Health and Disease (DOHaD) framework, which posits that environmental exposures during critical windows of prenatal and early postnatal development can permanently programme physiological systems [5–7]. Historical cohorts, such as those from the Dutch Hunger Winter, reveal that prenatal nutritional deprivation is associated with increased risks of cardiovascular and metabolic diseases decades later. Contemporary research further links early-life psychosocial stress to accelerated ageing in young adulthood [8]. Supported by studies linking early growth patterns and hormonal exposures to later-life mortality risks, these observations establish that developmental environments durably shape physiological trajectories related to health, disease and ageing [9–12]. By establishing a ‘molecular memory’ of early-life environments, the DOHaD framework provides a robust theoretical foundation for understanding how developmental conditions shape health trajectories and the rate of ageing [6,7].

Building on correlative human data, mechanistic studies in model organisms have solidified the concept that the perturbation of developmental signalling pathways often leads to delayed growth and extended lifespan. Pioneering genetic discoveries identified key evolutionarily conserved pathways, including insulin/IGF-1 signalling (IIS) in *Caenorhabditis elegans* [13,14] and *Drosophila* [15] and the growth hormone axis in dwarf mice [16]. This link between developmental physiology and longevity is further exemplified by the TOR pathway [17,18]. In these systems, mutations not only affect development and body size but also dramatically extend adult lifespan.

Crucially, emerging research using precise temporal control demonstrates that the longevity benefits of such interventions are strictly dependent on their application during development. For instance, transient inhibition of growth hormone action solely in the early postnatal period is sufficient to extend mouse lifespan [19]. Similarly, developmental delays induced through NF-κB inhibition in *Drosophila* [20], or through entry into larval diapause in the parasitoid wasp *Nasonia vitripennis* [21], confer long-lasting deceleration of adult ageing. Genetic manipulations that disrupt mitochondrial dynamics (*drp-1*) specifically during development can also profoundly modulate the longevity of *daf-2* mutants [22].

These findings converge on a powerful principle: developmental status determines longevity. The developmental phase is not merely a preparatory stage but an active programming period that establishes a long-term ageing trajectory. In this opinion piece, we provide a conceptual perspective on interventions confined to early developmental life, including metabolic stress (reactive oxygen species (ROS), mitochondrial stress, endoplasmic reticulum (ER) stress and dietary restriction (DR)), pharmacological agents (rapamycin), physiological factors (exercise) and ecological interactions (microbiome), that initiate enduring protective responses. By integrating evidence across species, we highlight how understanding developmental programming offers a unique strategy for promoting longevity and healthspan.

## 2. Developmental reactive oxygen species

ROS play a dual role in the ageing process. While excessive chronic ROS accumulation leads to oxidative stress and damage [23], moderate physiological levels function as crucial signalling molecules, generating beneficial effects through mitohormesis and adaptive stress responses [24]. A growing body of evidence, largely derived from *C. elegans* and *Drosophila*, suggests that ROS generated during development act not as toxic byproducts, but as pivotal signals that programme future stress resistance and shape adult lifespan trajectories [25–27].

In *C. elegans*, exposure to low concentrations of the oxidant paraquat during development extends lifespan through hormetic effects [25]. Parallel studies in *Drosophila* show that early-life exposure to mild oxidants (*tert*-butyl hydroperoxide) enhances longevity. However, through a distinct mechanism, rather than triggering a traditional long-term adaptive stress response, the oxidants act in an antibiotic-like manner to selectively deplete *Acetobacter* proteobacteria, resulting in a beneficial remodelling of the gut microbiome [27] (figure 1).

**Figure 1.**
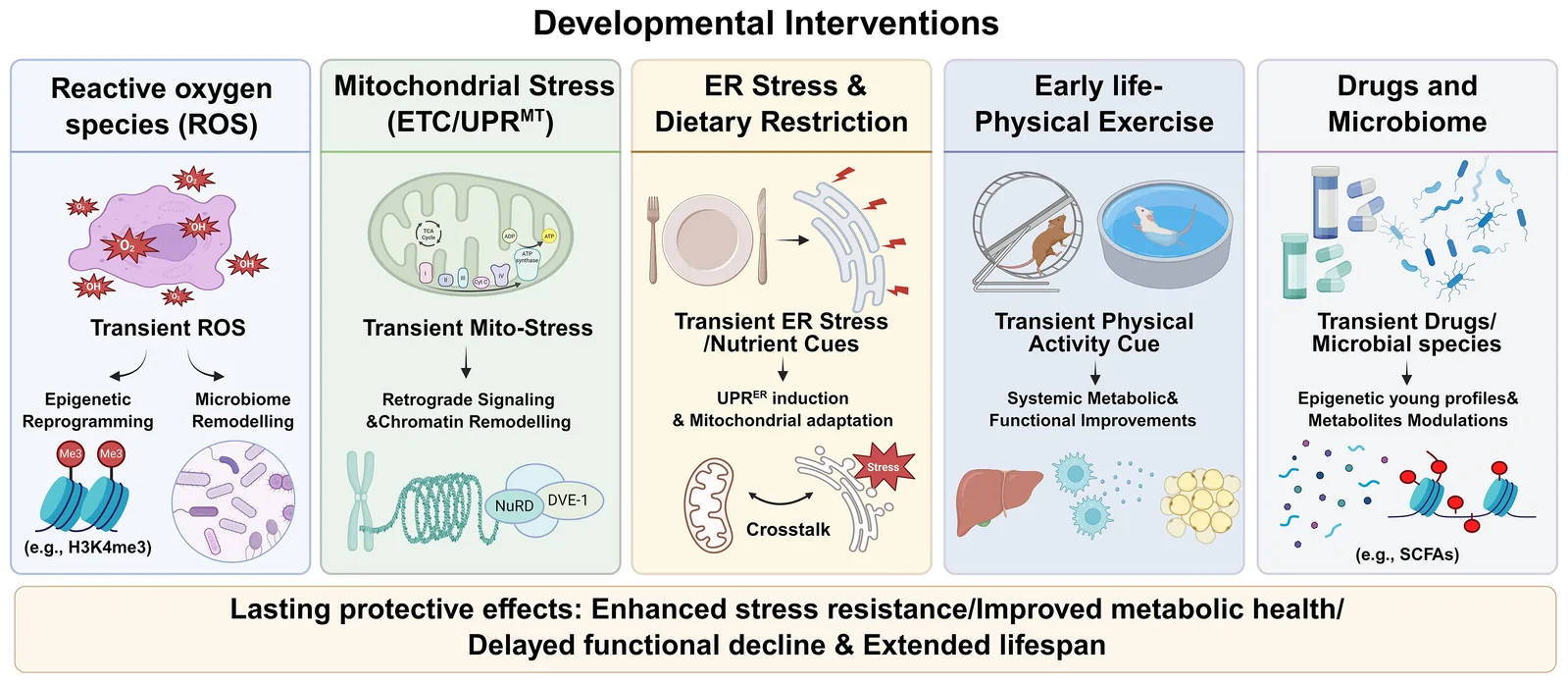
Transient developmental interventions induce long-lasting lifespan extension across species. Diverse stimuli applied during critical developmental windows initiate persistent pro-longevity programmes across species. These include developmental ROS, which can remodulate epigenetics or selectively remodel gut microbiota; developmental mitochondrial stress (e.g. electron transport chain (ETC) inhibition), which triggers nucleus-directed retrograde signalling and lipid metabolism; developmental ER stress and DR, which induce the ER unfolded protein response (UPR^ER^) or enhance mitochondria–ER contact sites (MERCS) and organelle communication; and other early-life physiological stressors, including exercise, transient rapamycin treatment or specific microbial exposure (e.g. *Mycobacterium* sp.).

The molecular basis of this programming is intricately linked to epigenetics [26]. Animals experiencing a developmental ROS pulse exhibit a cascade of long-lasting benefits, including enhanced stress resistance, improved redox homeostasis and a significantly prolonged lifespan [26]. Mechanistically, ROS inhibit the redox-sensitive SET/MLL (Su(var)3–9, Enhancer of zeste, Trithorax/Mixed-Lineage Leukemia) histone methyltransferase of the COMPASS (COMplex of Proteins ASsociated with Set1) complex. This results in a global reduction of the H3K4me3 epigenetic mark (figure 1), an altered chromatin state that reprogrammes gene expression to prime cytoprotective and stress-response pathways in later life [28] (figure 2).

**Figure 2.**
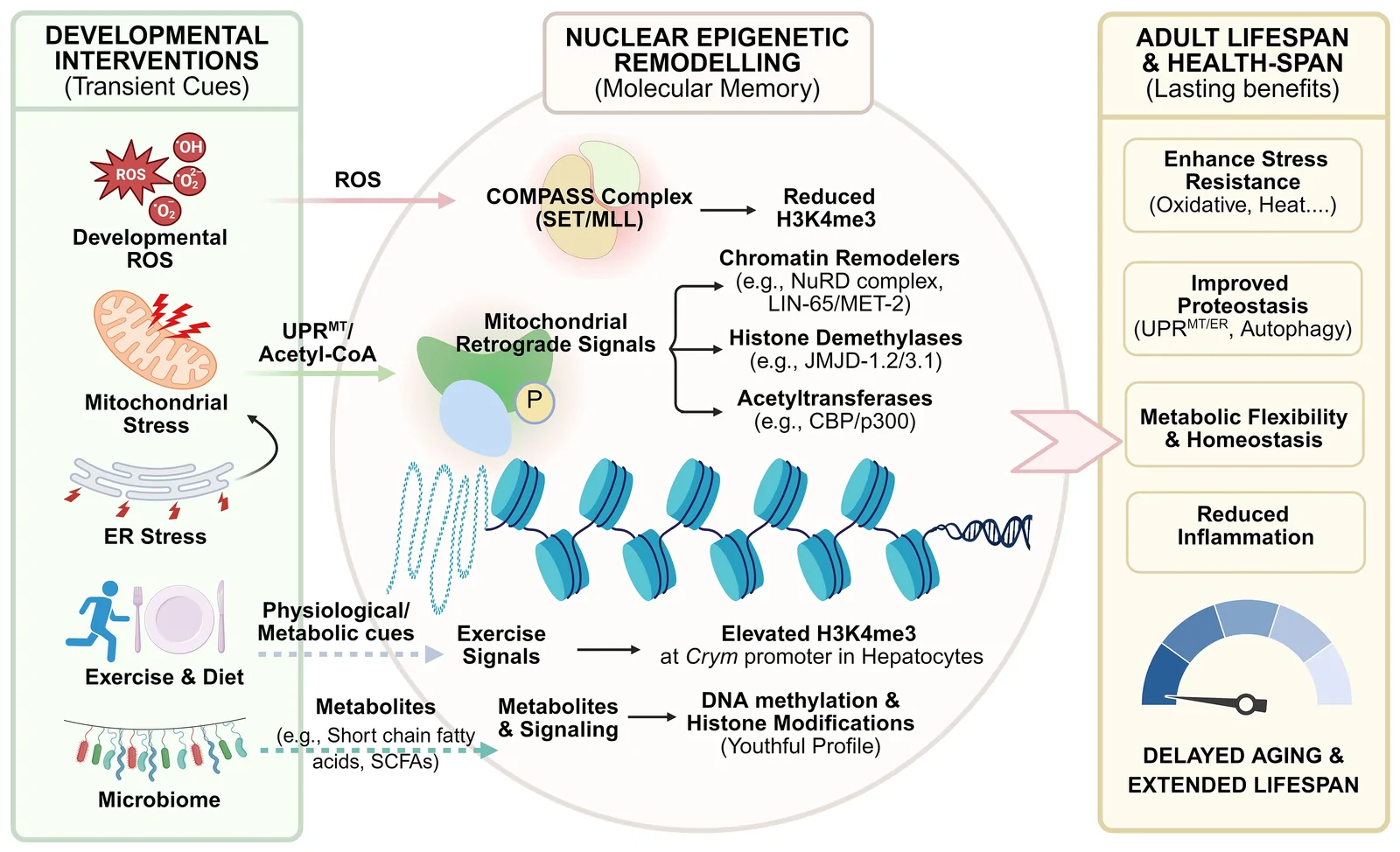
Enduring epigenetic remodelling is central to developmental programming of longevity. The long-term effects of transient developmental interventions are mediated by enduring epigenetic modifications that establish a molecular memory. Developmental ROS inhibit the COMPASS complex, thereby reducing H3K4me3 levels. Mitochondrial stress mobilizes a suite of epigenetic regulators, including histone demethylases (JMJD-1.2, JMJD-3.1), acetyltransferases (CBP/p300) and chromatin remodellers (nucleosome remodelling and histone deacetylase (NuRD) complex, LIN-65/MET-2). Early-life exercise specifically increases H3K4me3 at the promoter of *Crym* in hepatocytes. Similarly, rapamycin treatment or microbial metabolites potentially keep the youthful epigenetic profiles. Collectively, these changes establish a persistent, primed chromatin state that sustains enhanced stress resistance and metabolic homeostasis into adulthood.

## 3. Developmental mitochondrial stress and retrograde signalling

Perturbation of mitochondrial function during development can systemically influence the ageing process, a phenomenon validated across multiple model organisms [25]. Pioneering studies in *C. elegans* revealed that developmental suppression of electron transport chain (ETC) components through RNAi significantly extends adult lifespan, often concomitant with reduced body size and slowed behavioural rates [29–31]. Subsequent research in *Drosophila* confirmed that development-specific knockdown of ETC genes similarly prolongs lifespan [32]. Extending these findings to mammals, studies in mice show that loss of the mitochondrial protein SURF1—which perturbs ETC complex IV assembly—not only increases longevity but also confers remarkable resistance to age-related neurodegeneration [33] (figure 1).

A central mechanism implicated in this lifespan extension is mitochondria-to-nucleus retrograde signalling, which triggers profound epigenetic reprogramming [34]. Mitochondrial stress (e.g. ETC inhibition) during development induces the Mitochondrial Unfolded Protein Response (UPR^mt^). This is not an isolated organelle response; instead, it remodels the global chromatin state by releasing specific signalling metabolites (such as acetyl-CoA and ROS), thereby chronically altering gene expression profiles [35,36]. Several conserved epigenetic regulators execute this process (figure 2). Factors such as LIN-65 and MET-2 (homologue of the histone methyl-transferase SETDB1) are crucial for the chromatin reorganization triggered by mitochondrial stress [37]. Furthermore, histone demethylases JMJD-1.2/PHF8 and JMJD-3.1/JMJD3 activate stress-defence genes through demethylation [38], while the histone acetyltransferase CBP/p300 integrates mitochondrial stress signals to promote pro-longevity transcriptional programmes [39]. Notably, the Nucleosome Remodelling and histone Deacetylase (NuRD) chromatin remodelling complex has been identified as a central mediator, with its activity modulated by mitochondrial-stress-dependent changes in acetyl-CoA levels or phosphorylation of components like LIN-40/metastasis-associated factor 1 (MTA1) [36,40,41]. Together, these studies suggest the formation of a persistent chromatin landscape that may enhance stress resistance and ultimately promote lifespan extension (figure 2).

Furthermore, mitochondrial stress during development has been shown to safeguard nuclear envelope (NE) integrity and delay ageing via a redox-dependent lipid signalling pathway [42]. Specifically, developmental mitochondrial superoxide signals suppress SBP-1 (Sterol Regulating Element-binding Protein (SREBP) orthologue) activity, reducing unsaturated fatty acid biosynthesis and subsequent lipid peroxidation to preserve NE structure; notably, this mechanism appears conserved, as targeting lipid peroxidation ameliorates senescence in both human and primate cell models.

## 4. Developmental dietary restriction and endoplasmic reticulum stress

Complementing mitochondrial stress, compelling evidence indicates that mild, transient DR or ER stress experienced during critical developmental windows can extend lifespan in certain model organisms [43]. The core principle is ER hormesis, where a transient developmental stressor, such as nutrient scarcity, induces a persistent adaptive response centred on the Inositol-Requiring Enzyme 1 (IRE-1)/X-Box Binding Protein 1 (XBP-1) axis of the ER Unfolded Protein Response (UPR^ER^; figure 1). This early activation initiates organism-wide remodelling that sustains high-quality protein control and delays functional decline long after the initial challenge has passed.

Critically, this adaptive response possesses a pivotal mitochondrial dimension. The developmental UPR^ER^, particularly through its Protein Kinase R (PKR)-like ER Kinase (PERK) branch, acts as a long-range signal orchestrating organismal longevity [44]. This activation facilitates communication between mitochondria and the ER (figure 1), stimulating the formation of Mitochondria–ER Contact Sites (MERCS) [45]. This enhanced inter-organelle connectivity promotes mitochondrial capacity and mitophagy. Thus, transient developmental ER stress sets in motion a self-reinforcing cycle of organelle synergy, solidifying the foundation for energy demand and an extended lifespan.

However, while these inter-organelle synergies highlight the potential of developmental reprogramming in lower organisms, applying early-life DR paradigms to higher mammals reveals profound biological trade-offs. Studies in non-human primates demonstrate that the physiological impact of Caloric Restriction (CR) is highly complex and strictly dictated by the age of onset [46–48]. Crucially, implementing CR in very young primates can confer a significant survival risk [48]. Similarly, the optimal adult-onset CR can delay T-cell senescence, so these benefits are lost if the intervention is initiated during early development or late in life [49]. Consequently, extreme caution is required when considering the application of early-life caloric restriction.

## 5. Developmental exercise: physiological programming

A robust body of epidemiological evidence underscores the profound impact of physical activity initiated early in life. Higher levels of physical activity during childhood and adolescence correlate with improved long-term outcomes, including enhanced aerobic fitness, elevated bone mineral density and a reduced risk of metabolic and cardiovascular diseases in adulthood [50–52].

The mechanisms underlying this programming effect are being elucidated in animal models. Recent research in mice provides direct experimental evidence that early-life exercise is a potent intervention for healthspan extension [53]. A defined period of swimming exercise during youth (one to four months of age) in mice led to a significant healthspan extension, with a delayed age-related decline in physical function and reduced systemic inflammation, but without increasing maximum lifespan [53] (figure 1).

Mechanistically, early exercise induces persistent epigenetic modifications, specifically increasing H3K4me3 at the *Crym* promoter in hepatocytes [54] (figure 2). These modifications reprogramme cellular metabolism to increase the production of pipecolic acid, which exerts a significant anti-inflammatory effect maintained into middle age. Additionally, not limited to early exercise, it is well-established that physical exercise itself can induces extensive epigenetic modulations, such as DNA methylation, histone modifications and several miRNAs, thereby contributing further long-term effects [55–58]. In summary, these findings propose that physical activity may act as more than a temporary metabolic boost, potentially serving as a strategic investment in ‘epigenetic capital’ that compresses age-related morbidity.

## 6. Pharmacological and microbial interventions

Rapamycin is notable for its ability to extend lifespan across diverse species [59–62]. While effective in adulthood, its potential to extend lifespan through development-specific intervention has drawn significant attention [63]. Brief, early-life rapamycin treatment extends median lifespan and improves healthspan in male mice and *Daphnia magna* (figure 1). This treatment slows developmental growth and promotes lasting molecular rejuvenation, evidenced by a youthful transcriptomic and epigenetic profile that persists into old age [63] (figure 2). Supporting this, a separate study found that even a short rapamycin treatment in early adulthood confers lasting benefits, mechanistically driven by the sustained induction of intestinal autophagy [64].

However, these benefits entail significant trade-offs, as treated mice remained smaller throughout their lives and exhibited delayed reproductive maturation. Such phenotypes highlight the need for extensive safety considerations when modulating the mTOR pathway, which is essential for human growth and organogenesis [63,65]. Consequently, while rapamycin shows anti-ageing potential in older adults [66], its application during human development requires much more rigorous clinical evidence and careful evaluation owing to the potential risks of affecting essential growth trajectories [65,67].

Additionally, emerging evidence indicates that such long-lasting beneficial programming is also achievable through modulation of the microbes [68]. Evidence from *C. elegans* indicates that the soil bacterium *Mycobacterium* sp. (Root265) promotes health and longevity [68,69]. Notably, it was found that only developmental exposure to this bacterium was sufficient to extend lifespan, even if the bacterium was removed in adulthood [68] (figure 1). In mammals, maternal exposure to probiotics such as *Akkermansia muciniphila* enhances neurogenesis and gut stem cell function in offspring well into adulthood [70]. These effects probably operate through metabolites-mediated reprogramming, where microbial-derived short-chain fatty acids and amino acids modulate host mTOR pathways. Moreover, these metabolites are potent regulators of epigenetic machinery, potentially inducing lasting changes in DNA methylation and histone modification patterns in key tissues during sensitive developmental windows [71,72] (figure 2). These mechanisms collectively enable a transient early-life microbial encounter to exert a durable influence on the host's health trajectory.

## 7. Discussion and perspective

### (a) Enduring epigenetic remodelling as the core mechanism

The evidence synthesized here converges on a central mechanistic paradigm: transient physiological perturbations during development are translated into long-term health benefits through enduring, systemic epigenetic remodelling. This reprogramming acts as a molecular memory, setting a trajectory for enhanced stress resilience (figure 2). Whether induced by ROS, mitochondrial/ER stress, exercise or microbial metabolites, these interventions converge on chromatin regulation. This process is not a passive memory but an active, maintained state that enhances cellular proteostasis and metabolic flexibility throughout life.

### (b) Metabolic and cellular plasticity: defining the window of opportunity for anti-ageing

The efficacy of developmental interventions underscores a fundamental biological principle: developmental stages possess a unique degree of metabolic and cellular plasticity, allowing for durable reprogramming. However, this plasticity diminishes with age, defining a critical temporal window for intervention (figure 3).

**Figure 3.**
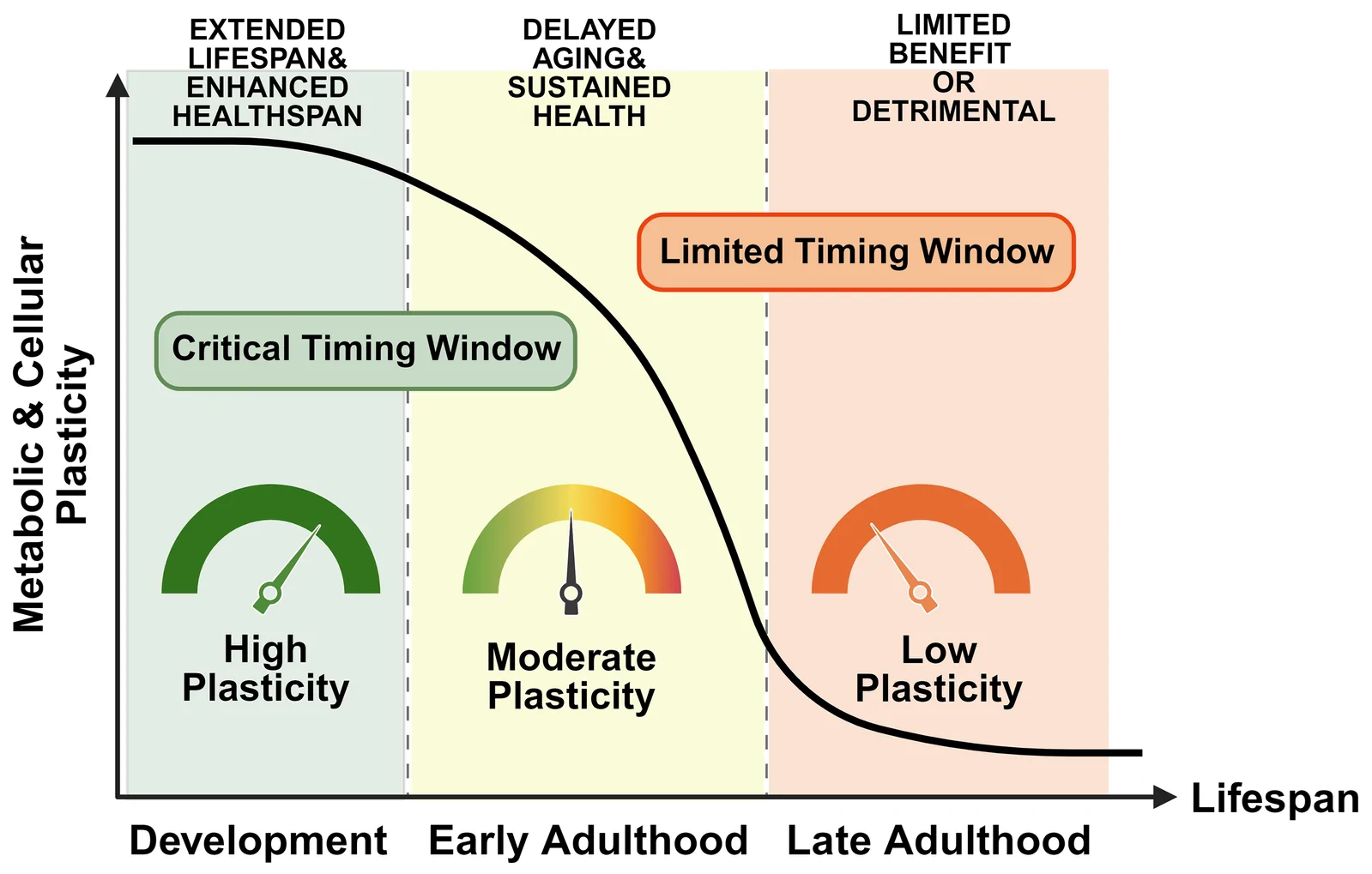
Metabolic and cellular plasticity defines a critical temporal window for anti-ageing interventions. The efficacy of interventions is highly dependent on the timing of application relative to the organism's life history. Developmental and early-life stages are characterized by high metabolic and cellular plasticity, allowing for durable adaptive reprogramming and lifespan extension in response to stressors. However, this plasticity declines with age. Consequently, the same interventions applied in late life may become ineffective or detrimental owing to compromised adaptive capacity and reduced metabolic flexibility.

Interventions targeting the mitochondrial ETC robustly extend lifespan when applied during development but often fail when initiated in adulthood [29,31]. Similarly, methionine restriction extends lifespan in *Drosophila* when applied in early adulthood but loses efficacy in late life [73]. Strikingly, some interventions exhibit age-related antagonistic pleiotropy: metformin promotes longevity in young *C. elegans* but becomes toxic in older worms owing to a loss of mitochondrial-mediated metabolic plasticity [74], and glucose restriction can shorten lifespan in young animals while extending it in aged ones [75]. These findings illustrate that the organismal response to a stressor is exquisitely dependent on the metabolic context of the life stage. The decline in plasticity involves the progressive attenuation of adaptive responses, such as the PERK-mediated UPR^ER^ [44]. Consequently, interventions that are hormetic during windows of high plasticity can become ineffective or detrimental in late life (figure 3).

## 8. Biological trade-offs, safety considerations and limitations

While shifting the geroscience paradigm towards early-life programming offers profound potential, realizing this translation requires careful navigation of the physiological and ethical challenges. Most notably, interventions that robustly extend lifespan when applied during development frequently carry significant physiological trade-offs [76,77]. For instance, developmental suppression of the ETC via RNAi, as well as early-life rapamycin treatments, are intrinsically coupled with delayed developmental growth, significantly reduced adult body size and slowed motility. Furthermore, classic genetic perturbations in highly conserved longevity pathways, such as the IIS and growth hormone axes, are also associated with retarded growth and often result in compromised reproductive fertility [14,19]. These pleiotropic effects suggest that the precise cues triggering enduring stress resistance and other benefits may simultaneously downregulate other critical energy-demanding processes.

Consequently, determining the feasibility of optimal timing and dosing remains a great challenge. Because developmental stages possess a uniquely high degree of metabolic and cellular plasticity, as we mentioned before, this threshold between a beneficial hormetic signal such as transient developmental ROS and irreversible pathological damage is extremely narrow. Inaccurate dosing or missing the right time frame could significantly disturb the key development instead of optimizing long-term health.

Furthermore, because most mechanistic research is currently limited to model organisms and small mammals, significant ethical constraints hinder direct human translation [78,79]. Applying systemic stressors or pharmacological agents to humans requires far more robust evidence from non-human primates and clinical cohorts, as adequate energy supply is vital for essential processes such as neurodevelopment and the maturation of the immune system. Consequently, the immediate value of studying early-life interventions lies not in direct treatment, but in decoding the precise molecular memory and enduring epigenetic remodelling they establish. By understanding these chromatin states, we can develop safe mimics that trigger youthful plasticity in adults, securing long-term health benefits while bypassing the inherent risks to development.

## 9. Conclusion

This perspective proposes that the traditional ‘damage-repair’ model could be complemented by a ‘programming’ framework focused on early-life plasticity. While evidence from model organisms suggests that the most potent anti-ageing interventions may be most effective when applied during windows of high plasticity such as development, adolescence or early adulthood, their potential translation to humans necessitates a rigorous evaluation of biological trade-offs and safety. Further research must map these critical windows with precision and explore methods to safely ‘reopen’ plasticity in adulthood while addressing the ethical challenges of early-life interference. By doing so, we can provide a valuable conceptual framework for exploring future strategies to delay ageing.

## Data Availability

This article has no additional data.

## Theme

One contribution of 21 to a theme issue ‘Developmental circuits in ageing’.
